# 

**Published:** 1872-07

**Authors:** 


SAVANNAH MEDICAL COLLEGE.—We acknowledge the receipt of the announcement of the Savannah Medical College for the session of 1872–'73, under new arrangements, in connection with improved hospital and clinical facilities, which, we shall be pleased to know, will be attended by an increased and increasing prosperity.

BOOKS, etc., received, will be noticed in the next number of the JOURNAL.

